# Coaching past simple forms of habitual expressions beyond the textbook: A corpus-based experimental investigation

**DOI:** 10.1371/journal.pone.0346593

**Published:** 2026-04-28

**Authors:** Aman Matebie Dagnaw, Abiy Yigzaw Filatie, Ebabu Tefera Adugna

**Affiliations:** College of Education, Department of English Language Education, Bahir Dar University, Bahir Dar, Ethiopia; UKM: Universiti Kebangsaan Malaysia, MALAYSIA

## Abstract

The present study was a nonequivalent pretest-posttest control group design investigating the effects of corpus-based instruction on grammatical accuracy and written application of past simple forms of habitual expressions. An experimental group was exposed to two weeks of corpus-based instruction as compared to control group that had been exposed to a conventional grammar instruction. Quantitative and qualitative analysis of written tests, along with comparisons of Ethiopian Students Corpus to the British National Corpus was carried out. Common learner mistakes, including subject omission, misuse of used to, and overextension of would to single past occurrences were identified. The two groups showed improvement on the post-test, but the group taught through corpus-based instruction demonstrated significantly stronger improvement and a smaller number of errors: – overgeneralisation and subject-verb agreement issues were observed. This showed that the corpus-based instruction can be used to make accurate and contextually appropriate command of past simple habitual constructions in writing despite the small technological drawbacks.

## Introduction

Grammatical correctness is one of the foundations of assessing the writing skills due to its implication of dramatic significance on the academic development of learners in the context of the higher education [1,2]. Although it can be stated that greater concepts such as paragraph structure and ideational unity are more relevant [3], grammatical correctness is also an essential background. Therefore, a good grammar is clear and concise in order to be able to convey ideas.

Studies such as those by [4,5] have recommended that the finer aspects of writing, such as the tense of verbs and agreement may happen to reveal a lot particularly when comparing the two groups of non-native and native speakers. Such grammatical complications can be used to determine the areas where students may be weak especially in tense/agreement (T/AGR) inflection and the passive voice [5]. Considering these particular difficulties, teachers will be able to provide students with the necessary instruments that will help them to become good and respected writers.

Although there are those who support the idea of de-emphasizing grammar in the favour of the expanded writing skills [6,7], the method can provide the inherent relationship that exists between grammatical word and mean in written communication [8]. Good teaching grammar extends beyond memorization; it also enables learners to comprehend the way language works in producing the best and effective ways of passing the ideas across. By so doing, the process of teaching grammar will be part and parcel of the writing process aiding students at every level of brainstorming through to the final editing.

Considering the above discussion, the emergence of the corpus linguistics has had significant implications in the study and teaching of languages. To a great extent, this change is predetermined by the peculiarities of corpus data like authenticity, verifiability, and enormity [9]. Corpus linguistics is a usage-based language teaching/learning method since any study/teaching practice that consists of a corpus is based on recorded data- actual usage of a type/genre of language [10]. It is a general theory of language and fundamentally serves as a prism to predict the language. It is achieved by using text-based methods to ensure that language learners refer and get answers in the real-life use [11] to guide further choice concerning teaching, learning and using language.

In actual sense, the corpus is a frame line of writing, which forms part of an exercise in every spoken and written composition [12]. Among others, the most representative corpus used in English language teaching (ELT) is the British National Corpus (BNC) that provides numerous opportunities such as searching suitable word forms and search of word form groups. Corpus helps the students with every step of improvement. As an example, during the pre-writing stage, it hypnotises them to come up with ideas on the subject they are going to write down.

The research, thus, based its balance, generality and suitability on British National purpose (BNC). To begin with, it is widely believed that it is a balanced Corpus- BNC model has been adhered to when a number of corpora have been constructed (see chapter Two). Second, it is a general corpus (British English type), and it was appropriate to make mini materials to be handed out to students at grade eleven who were studying English as a General purpose (EGP). EGP is designed to equip the students with the preparation to communicate to the target language in general circumstances [13]. Third, being more of written corpus, it appeared to be true that BNC exhibits numerous grammar construction concordance traces in written genres. It is then compared to the objective of this research study that will deal with grammatical correctness with reference to writing skills.

It was generally believed that, by incorporating the discoveries of corpus linguistics into grammar pedagogy, it becomes a significant aspect of grammar instruction and pedagogical resources in EFL situations where such instructions and materials are comparably the same over a long period of time [14]. In addition, the revolutions occurred in the grammar teaching accompanied by advantages of corpus application in EFL situations (printing mini-texts).

### Statement of the problem

The advent of corpus linguistics in the late 20th century has changed the paradigm of grammar study whereby the focus is made on register specific description, grammar-vocabulary interface, and grammatical structures in contextual situation [15]. However, it appears that Ethiopian EFL teachers are not using the opportunities. The corpus-based information should be incorporated into the English textbooks in the secondary school because it will allow building the linguistic competence and flexibility of the students in the real world situation.

Better still, recent publications on the teaching of grammar have examined the significance of contemporary teachers of English to adopt a teaching methodology that will cultivate in the students an awareness of the grammar in context. It is an effective demand that researchers have been attempting to redress [16–18]. Nevertheless, despite the improvement, some more work is required.

Several barriers to the use of the corpus methods by Ethiopian teachers exist. This is due to an overall deficiency in the natural language exemplification and the interpretation of corpus, which often results in the inability to match prescriptive grammar and natural usage [19]. This discontinuity puts a constraint on the ability of learners to know contextual word usage, such as examples given by BNC concordance lines of frequencies of present tense use of the word among native speakers, L2 learner and between registers, e.g., academic text, news, fiction, and conversation. To address these problems, one can learn grammar with the help of technological resources that are user-friendly.

These issues are also noted in the studies of EFL students in other countries. [20] also found that the learners with ESL in Malaysia were experiencing issues with tense and agreement morphology due to the low exposure and comprehension of the tense rules. [21] noted the influence of native languages (Malay and Chinese) on the learning of tenses which were not easy to learn even among the able students. The same case studies need to be conducted on Ethiopian students.

The special difficulties of past-simple expressions of habitual are due to the primary place of such expressions in the framework of communicative semantics and aspectual interpretation [22,23]. The habitual meanings in the past can be expressed using a wide range of grammatical and lexical forms, such as the past simple tense form itself, frequency adverbials and periphrastic clause. In contrast other grammar features, the past simple expresses both eventive (single, completed) and habitual (repeated) interpretations and this frequently causes confusion and is commonly abused by learners of second language. It has always been demonstrated that learners are inclined to overgeneralize the eventive role of past simple and underutilize or misinterpret the habitual one [24,25]. It is marked by these problems, especially in verb phrase realization and sentence construction [6], and Ethiopian EFL learners are especially prone to such mistakes in the process of trying to convey habitual past events in an appropriate way.

The general evidence of underuse, overuse, and excessive use of temporal adverbials to compensate lack of grammatical control has been found in studies on a wide range of learner groups, including Asian [25–27], French [28], and Chinese learners [29,30]. Students tend to not master traditional form/meaning combinations and are subject to the L1 transfer effects, primarily in the repeated actions which are said in different ways or words in the native language. These problems are further complicated by pragmatic and cultural influences to the interpretation of the repeated past events. As corpus-based study has revealed, the most common realisations of habitual past meanings in natural discourse are based on the simple past with frequency adverbials [1,31], and corpus-based teaching has been found to be effective in increasing the awareness of the learners regarding the patterns of usage, frequency of use, and the contextual restrictions [15,32]. Regardless of such developments, there is a research gap that has not been filled through any empirical studies on the use of past simple habitual expressions by Ethiopian EFL learners and this is where the current study will fill the gap.

Corpus-based instruction (CBI) is fruitful as demonstrated by the international studies. [33] probed that the effect of CBI homework materials improved the comprehension of post-predicate -ing forms/infinitives among EFL learners, particularly in resource-starved settings. The performance and motivation of Chinese undergraduates were also improved which [34] demonstrated, but [35,36] also mentioned the improvement in lexico-grammar, critical understanding, and discovery learning. The other studies [37–40] acknowledged that both the direct and indirect techniques of corpus are superior to the traditional techniques.

Nonetheless, there are still issues. [41–44] caution that, untrained teachers and students may not have an incentive to make use of corpora. [45] used to mention that corpus tools were not known to learners and their focus was on exams and [46] argued that corpus applications can be used better with specialists than with classroom teachers, which can limit interaction.

Even the teaching of grammar in writing in the L2 language remains a still debated topic, both in terms of its volume [47] and efficiency [48]. The traditional approach is more focused on the actuality rather than communicative application, but it has been proven to be different with a sense-making grammar instruction on meaningful writing activities that enabled more interest [49].

Most of them, unlike CBI where it is often preferred in international studies, are not specific on rates of accuracy, effect sizes, or rigor in their procedures, they are more likely to employ p-values or general statements. The researcher has not found any studies concerning the effect of CBI in written grammatical correctness of learners in Ethiopian secondary schools as far as past simple expressions on habitual basis are concerned. It is a gap that is worth being studied given the inconclusive results of cross-border CBI studies. The current research is therefore founded on this that the current research investigated the impact of CBI on the written grammatical accuracy of the Ethiopian students on the past simple forms of the habitual expressions. So, this research aspired to answer the following questions.

1. Is there significant mean score difference in appropriate use of Past simple forms of habitual expressions between experimental and control group students
2. What are the frequency distribution of past simple forms of habitual expressions in both the Ethiopian Students Corpus and the British National Corpus?
3. What are the error patterns of past simple forms of habitual expressions in both Experimental and control groups?

## Materials and methods

### Study design

The study was conducted using a quasi-experimental research design namely a nonequivalent pretest- posttest control group design to investigate the effects of corpus-based instruction on the grammatical correctness of the modal auxiliary verbs of ability among students. In order to manage any potential confounding factors that could be brought about by the fact that the participants were not selected randomly, the study had a comparison group that was very similar to the experimental group. Of importance is not only whether the experimental group improves, but also whether they do so more than the control group improves.

### Context and participants

The participants of the study were grade eleven students of Ethio-Japan Secondary School in Bahir Dar Town in Ethiopia. They were between the ages of 17 and 20 years with 51 females and 44 males and a total of 95 students. Students were all of the same first language Amharic, and had only been exposed to English in formal education. English was studied as a subject, and was also the language of instruction of the other subjects at their level. Although there were a few observable mistakes and challenges, the students could understand and engage in oral and written exercises in English.

Two Grade eleven sections of this government school were selected out of the six sections through a random sampling method. The undertaking of this random selection process was also applied in assigning one group as experimental group and the other as comparison group. The pre-test was done to the two groups. The purpose of this pre-test was to make sure that the students in either of the groups were similar in the way of using past simple forms of habitual expressions. This assists the researchers to verify that the observed variations in the results might have been a result of the instructional intervention as opposed to the existing variations between the groups.

### Target grammatical feature

The target grammar structure, past simple forms of habitual expressions was selected on the basis of the teaching experience and curriculum. Teachers discovered that learners have a difficulty in the use of this feature. Also, students abnormally use forms of past simple of habitual expressions or misuse them due to their lack of knowledge about their register-specific applications and sentence structures [50,51]. It is also a subject where students require further assistance as the Grade Eleven English textbook used in Ethiopia also focuses on past simple forms of habitual expressions. The attention to this feature can solve these issues and can be attributed to the priorities of the curriculum.

### Corpus compilation and intervention

To overcome the lack of an appropriate and appropriate corpus-based instruction material (Corpus- informed teaching material) to use in the experimental group, researchers referred to the works of [19,52–54]. The lesson plan was made classroom-based and self-study tool of learning. To design corpus-informed materials to be used in the intervention, there was a sequence of steps to follow. At first, a particular corpus was selected (British National Corpus BNC 2014) due to the fact that it is a general corpus. Subsequently, the BNC written genre was used to produce concordance lines on the tool of Sketch Engine Language Learning (SKELL). These concordance lines were, then, edited manually to remove any line with difficult grammatical patterns. This makes sure that the material was appropriate to the students. Lastly, exercises were made to guide the students to recognize and apply grammar patterns within a context.

The teaching material based on corpus was simple and presented grammar contents in clear charts. They were included on the grammar of written English as it appears in high school teachers, textbooks, academic essays, high school classrooms, and dialogues between teachers and students. The target constructions that are represented in these charts have been re-tested using actual real-life data of findings of corpus-based research. It is in order to make sure that they are an authentic representation of particular registers. In the corpus-informed material, many of the grammar presentations and application sections were presented. These have a feature named ‘Data from the Real World’ in which real and useful points are found by analysis of corpus data. Also, the section of ‘An Avoid Common Mistakes’ can be used to cultivate students’ awareness that includes the most typical mistakes that English Language learners make. In addition to this, it enables them to train in noticing and correcting these mistakes in textual running. This part can assist the students to prevent such errors in their work. The errors presented in this part were based on real academic data on student corpus, essays by learners who did not learn English as their mother tongue, and the opinions of classroom teachers.

On the other hand, the conventional grammar-teaching procedures were utilized with the control group. The teaching material was English for Ethiopia Student Textbook Grade Eleven (1st edition, MoE, 2023). The Grade Eleven textbook of this group contained certain grammatical explanations, examples, and wide language exercises. As an example, a note on past simple forms of expressions of habitual modal auxiliary (Used to, would) are added providing their explanation of when to use to express past habit.

The objective of the intervention was to compare the results of teaching the students with the use of corpus to teach grammatical correctness in past simple forms of habitual expressions. Prior to the experiment, the researchers referred to various web sources and tutorial videos on corpus use primarily BNC2014 to analyse frequent and erroneous words. Subsequently, mini-texts formation on the basis of concordance lines were prepared. In the course of the intervention, leading the students to grammatical patterns, performing grammar exercises and using corpus-based materials to write compositions, respectively, were the most important actions.

To be more precise, the corpus of learners was designed on the basis of Paragraphs written by grade 11 students Ethiopian Students Corpus. They had to compose a paragraph on what they were instructed: write a paragraph about what you did and did not do often in the past and compare it with what you do and do not do now. The contents of the compositions of the participants were translated into text files to obtain corpus data. The data was purged and subsequently posted on the SKELL web site as the Ethiopia Students Corpus. This formed the primary collection of texts to be used in frequency and error analysis in comparison to BNC2014 used as a reference corpus. Ethiopian Students corpus is made up of 33, 271 running words.

The intervention period was two weeks and began on 17/09/2025 and concluded on 12/10/2025 excluding home take work. It was involved in the instruction of past simple forms of habitual expressions and training of the same using corpus-based teaching and students were involved in a number of activities. As noted above, they started recognizing and studying actual examples of past simple forms of habitual expressions by a corpus to find out common use and context. They used the expression in affirmative, negative and interrogative forms using used to, would and verb 2, and analysed data to determine the most frequent and the least frequent expressions. The experimental group was then directed to the practice of preventing general grammar errors that are related to this target grammar constructions.

### Writing tests

The writing test had multiple-choice test, gap-filling and paragraph writing. The test had ten items to be rated. In the multiple choice questions, the students were required to pick the most appropriate among the available alternatives. In paragraphs where verbs could be filled in by the students with the right verb forms (used to, would and verb 2 forms), the verbs were written in bracket. In the writing paragraph part, the participants were subjected to compose an essay comparing their previous habits.

The two-phase writing tests were some of the measures used to determine the past simple forms of the habitual expressions usage and grammatical accuracy of the participants in an experimental study on the effects of a particular intervention. Pre-tests gave the baseline competency whereas post-tests measured the impacts of the intervention. Based on the already existing research papers [55], the participants were asked to write 7–11 line paragraphs with the same instructions and time constraints on both test phases. Word limits (50–70) and time (45–50 minutes) time limits were the same. Adapted standardized marking criteria that used [56] ensured reliable assessment in the pre-and post-test. The purpose of this writing test and scoring rubrics was to give a clear picture of the intervention as being effective in enhancing efficacy of the participants in mastering past simple forms of habitual expressions and general grammar.

The consistency of the tests was determined by administering the test to another sample that was not that of the current research. The test-retest reliability coefficient of the past simple forms of habitual expressions test was identified to be 0.78 that depicts a high degree of reliability. In addition, the tests were validated by analysing them by TEFL experts.

### Analysis

Corpus-based instruction was used to teach the role of the past simple forms of habitual expressions in terms of their application by the students using quantitative data analysis and corpus analysis. In the case of the quantitative analysis, the independent and paired-sample t-tests were employed to compare two group performances in terms of data gathered in the form of written tests. These exams comprised of three parts the multiple-choice questions, gap-fill-in questions, and the writing tasks. The multiple-choice and gap-filling parts were analyzed using independent and paired-sample t-tests, whereas the paragraph writing was evaluated with the help of corpus analysis (frequency distribution and error analysis).

The analysis of corpus was done in three steps. The student texts were firstly converted into text files. Second, the texts have been added to the Sketch Engine of Language Learning (SKELL) that created error codes. Some of the mistakes that were detected were the misuse of used to, would and Verb-2 ambiguity in past simple forms of the habitual phrases, and the common grammatical mistakes, e.g., subject-verb agreement and verb usage. There was categorization of errors according to a standard error coding scheme. Lastly, the past simple forms of habitual expressions was captured.

### Ethics statement

It is notable that informed consent is one of the Ethical issues that will be taken into consideration in this study. The Ethical research involves informed consent [54,57]. To this end, the purpose of the study, the type of data to be gathered among the participants, the degree of commitment that the participants were required to give, and the possible risks of the participants involved in the study were made clear to the participants of the study. Moreover, the right to withdraw consent without any reasons and any time, the right to the confidentially of the identity of the participant, the clarity about the management of data and the right to get additional information were considered.

To make it more specific, following the IRB approval, the researchers had a meeting with the school principals of Ethio-Japan Secondary School, the heads of English Language departments and English Language teachers. Once the institutional agreement was established, the students received the information regarding the purpose and procedures of the study and requested their voluntary involvement. The participants were given informed verbal consent in which they were informed of the involvement and formally signed this consent and documented it in the school records.

The other concern that was brought up in this research is the principle of justice. As noted in the above section, this research had to have two groups; experimental and control. Corpus-based instruction, which is a relatively recent instruction, was used to teach the experimental group, and not to teach the control group. In fact, despite the random selection of the groups, one can argue that the experimental group could use the intervention which would lead to certain changes in grammatical construction learning. A control group also received such training and exercise on CBI in order to offset the advantages which were received by the intervention.

### Consent of ethics committee

The research was also established to have been reviewed and given the ethical clearance of Humanities Faculty Research and Ethical Clearance Committee of Bahir Dar University, Ethiopia. The research was conducted in compliance with the ethical principles which guarantees the confidentiality of the participants, their voluntary participation, and the data security. The ethical standards were observed in the research in the course of the research work, and ethical approval was taken before the study started.

## Results and discussion

### Statistical analysis of written tests

#### Independent samples t-test for past simple forms of habitual expressions usage.

According to Table 1, the independent samples t-test results showed no significant difference in Past simple forms of habituals between the control and experimental groups on the pre-test. The t-value was 1.39, with a p-value of.890 (p > 0.05), indicating that the difference in mean scores between the groups was not statistically significant. This suggests that any observed differences could be due to chance, aligning with the descriptive statistics.

**Table 1:** The pre-test results of statistical analysis of independent samples t-tests.

Grammar	Group	N	Mean	SD	T	Df	Sig.(2-tailed)
**Past simple tense**	Control	48	8.10	3.562	1.39	93	.890
	Experimental	47	8.21	4.054			

After analyzing the pre-test results and observing no significant differences between the control and experimental groups, the study proceeded to investigate the effect of corpus based instruction on post-test results.

As shown in Table 2, the inferential statistics of the post-test results on Past simple forms of habitual expressions revealed a significant difference between the control and experimental groups. The experimental group had a higher mean score (M = 12.11, SD = 3.205) compared to the control group (M = 8.21, SD = 3.402). This difference in performance was statistically significant, as indicated by the t-test results (t (93) = 5.746, p = .001). These findings suggest that corpus based instruction had a positive impact on the experimental group’s ability to use Past simple forms of habitual expressions as their scores were significantly higher than those of the control group.

**Table 2:** The pre-test results of statistical analysis of independent samples t-tests.

Grammar	Group	N	Mean	SD	T	Df	Sig.(2-tailed)
Past simple tense habitual expression	Control	48	8.21	3.402	5.746	93	.001
	Experimental	47	12.11	3.205			

#### Paired samples t-test for past simple forms habitual expressions usage.

A paired sample t-test was employed to analyze the changes within each group to measure the improvement or lack thereof resulting from corpus-based instruction.

As shown in Table 3, the paired sample t-test results for the experimental group indicated a statistically significant improvement in students’ use of Past simple tense habitual expressions after being taught through corpus-based instruction. The mean score for the “choice “was 10.87, SD = 1.94. The total mean score was 20.32, SD = 3.04. The t-value for this group was 8.76, with a p-value of 0.001, < 0.05, indicating that the differences observed are highly significant.

**Table 3:** Paired-samples t-test results on Past simple tense forms of habitual expression usage.

Groups	Grammar items	N		Paired Differences					
			Mean	SD		Df	95% confidence interval		Sig. (2 tailed)
							Lower	Upper	
**Experimental**	choice	47	10.87	1.94	8.76	46	−4.77	3.00	0.001
	Gap		9.4	2.15					
	Total		20.32	3.04					
**Control**	Choice	48	7.63	1.60	.289	47	−0.82	.620	0.774
	Gap		8.69	2.06					
	Total		16.31	2.49					

In contrast, the paired sample t-test for the control group, which was taught using a conventional grammar teaching method did not show a significant improvement in the students’ use of Past simple tense clauses. The mean score for the “choice” grammar item was 7.63, SD = 1.60, and the total mean score was 16.31 with SD = 2.49. The t-value was 0.289, and the p-value was 0.774 which is much higher than the typical alpha level of 0.05 which indicates that the differences before and after the instruction are not statistically significant. These results suggest that the conventional teaching method did not significantly enhance students’ past simple tense forms of habitual expressions as effectively as the corpus-based instruction used with the experimental group.

Additionally, Cohen’s *d* was calculated to determine the effect sizes for both the experimental and control groups. The experimental group showed a Cohen’s d value of 0.72, which corresponds to a medium to large effect size. This showed a substantial improvement in students’ use of past simple habitual expressions. In contrast, the control group exhibited a Cohen’s d value of −0.57. Ignoring the negative sign, this still represents a medium effect size, although it reflects a decline in performance. These effect size levels, based on the thresholds of small (0.2 ≤ *d* < 0.5), medium (0.5 ≤ d < 0.8), and large (d ≥ 0.8), emphasize the greater practical impact of corpus-based instruction over conventional grammar teaching methods.

### Frequency distribution of past simple forms habitual expressions

The past simple tense is a core grammatical resource in English for expressing completed actions, states, and habitual events in the past. Among its principal realizations, used to, would, and the simple past verb form (V2) are central to describing repeated or customary past actions [58]. Examining the frequency and distribution of these constructions across corpora provides insight into differences between native speakers and second language learners. The present study compares the Ethiopian Students Corpus and the British National Corpus (BNC 2014) to identify patterns in past simple habitual usage.

Because the Ethiopian Students Corpus (53,214 tokens) is substantially smaller than the BNC (110,723 tokens), frequency normalization was applied to allow valid comparison. Normalized frequencies per 100,000 words were calculated following standard corpus-linguistic practice [1].

**Fig 1:**
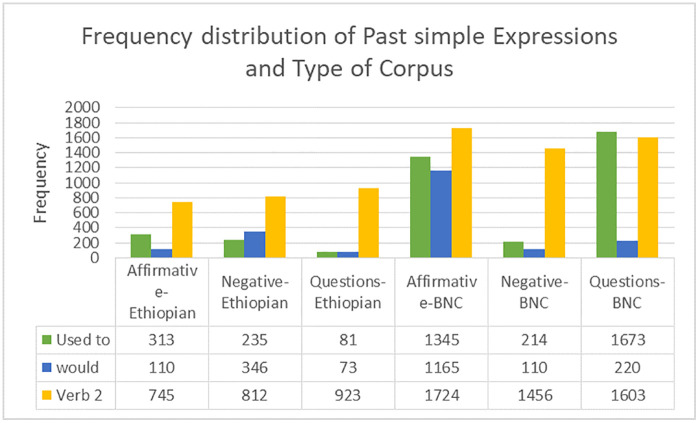
Comparison of frequency of Past simple expressions in Ethiopian Students Corpus and British National Corpus (BNC 2014).

Fig 1 illustrates the distribution of used to, would, and V2 forms across affirmative, negative, and interrogative constructions. Generally, Ethiopian learners use past simple habitual expressions less frequently than native speakers with particularly limited use in interrogative contexts.

#### Use of used to.

The Ethiopian Students Corpus shows substantially lower frequencies of **used to** across all clause types, especially in questions. While affirmative and negative uses are present, interrogative forms are rare in comparison with the BNC. This tendency is reflected in learner production, where students typically avoid forming questions such as “Did people use to communicate by letters?, favoring affirmative statements instead.

In contrast, the BNC contains frequent interrogative uses of **used to** which demonstrates native speakers’ flexibility in manipulating this structure. This discrepancy may be explained by limited opportunities for meaningful output in EFL contexts. Swain’s Output Hypothesis [58] suggests that insufficient production practice restricts learners’ ability to control complex grammatical forms, particularly those involving auxiliary inversion.

#### Use of would.

Distinct distributional patterns also emerge in the use of would. Ethiopian learners overuse would in negative contexts while underusing it in affirmative and interrogative forms relative to native speakers. For example, learner data include constructions such as “People wouldn’t communicate easily before phones”, where would is preferred over a simple past form.

Native speakers in the BNC, however, employ would more frequently in affirmative narrative contexts to describe past habitual actions, such as “We would gather every evening to watch television.” The imbalance observed in learner usage may result from classroom-driven instruction, where modal verbs are taught explicitly and often associated with restricted functions. As [59] notes, EFL learners frequently overgeneralize forms that are explicitly taught that to non-native-like frequency patterns.

#### Use of Verb2 (Simple Past Form).

The simple past verb form (V2) occurs more frequently in the BNC across all sentence types. The largest difference appears in affirmative constructions which shows that Ethiopian learners may rely on a narrower range of past-tense forms in extended discourse. For instance, learners often produce short, formulaic sentences such as “People studied in libraries before the internet”, while native speaker data show more sustained narrative use of past tense verbs.

Nevertheless, Ethiopian learners display relatively high frequencies of V2 forms in interrogative contexts, as seen in examples like “How did people learn before online courses?” This pattern likely reflects pedagogical emphasis on question formation in EFL classrooms. In this regard, [56] argues that learners’ tense–aspect development is strongly shaped by instructional focus, particularly in contexts with limited natural exposure.

To determine the proportional frequency of each past simple tense construction, the following formula was applied:


$$\text{NF}=\frac{\left(Number\;of\;occurrences\;of\;the\;construction\right)}{Total\;size\;of\;the\;corpus}\;\times \;\text{Normalization base }(\text{e}.\text{g}.,\text{ 100},\text{000})$$

Normalized frequencies in Table 4 provide a more accurate representation of cross-corpus differences. Although raw frequencies suggest substantial disparities, normalization reveals proportionate contrasts that are genuinely linguistic. For example, affirmative used to occur 588.19 times per 100,000 words in the Ethiopian corpus compared to 1214.74 in the BNC. Similarly, while would appears less frequently in Ethiopian affirmative contexts, normalized results confirm systematic differences rather than artifacts of corpus size. These findings demonstrate the necessity of normalization in corpus-based comparisons.

**Table 4:** Frequency normalization table (per 100,000).

Type of Corpus	Affi mative (used to)	Negative (use to)	Questions (used to)	Affirmative (would)	Negatie (would)	Questions (would)	Affirmative (Verb2)	Negative (Verb2)	Questions (Verb2)
Ethiopian Students	588.1	441.6	152.22	206.71	650.2	137.18	1400.01	1525.	1734.5
British National Corpus	1214.4	193.2	1510.9	1052.18	99.35	198.69	1557.04	1314.9	1447.7

### Error patterns in past simple forms habitual expressions

The post-test writing task required participants from both control and experimental groups to describe changes in daily life brought about by technology, using used to, would, and simple past verb forms. Analysis of the scripts revealed recurring error patterns, with the control group consistently producing more errors than the experimental group.

#### Subject omission in time clauses with “Would” or “Used to”.

Subject omission emerged as a frequent problem, particularly in the control group. Learners often failed to maintain subject continuity across clauses, resulting in ambiguity. For example:

“Before smartphones, people used to write letters and waited many days for replies.”

In this sentence, the subject of the second clause is implicit rather than explicit. A clearer version would be “people used to write letters and they waited many days for replies.” The experimental group demonstrated greater consistency in maintaining explicit subjects that indicates improved syntactic awareness.

#### Common omission of “-d” in affirmative forms of “Used to”.

Omission of the -d in affirmative used to constructions was one of the most frequent errors. Control group learners regularly produced sentences such as:

“People use to write letters before email.”

In contrast, experimental group learners more consistently produced correct forms, for example: “People used to write letters to communicate.”

The reduced error rate in the experimental group suggests that exposure to authentic corpus examples supported the internalization of correct morphological patterns Incorrect Usage of “Use to” in Negative Statements and Questions Involving “Did”

Another recurrent error involved incorrect retention of used to in negative and interrogative constructions with did. Typical control group examples include: “People didn’t used to communicate online.”

“How did people used to learn before the internet?”

These errors reflect incomplete understanding of auxiliary verb behavior, as did requires the base form use. The experimental group produced fewer such errors, indicating that corpus-based instruction helped learners recognize and apply the correct structural pattern.

#### Improper use of “Would” in singular past events.

Learners in both groups occasionally misused would to describe single, non-habitual past events. For instance: “People would access the internet for the first time in 1995.”

Here, would incorrectly implies repeated actions whereas a simple past verb (accessed) is required. Although such errors occurred in both groups, they were notably fewer in the experimental group which suggests improved sensitivity to the contextual constraints governing would.

## Discussion

The findings of the study which aimed to explore the effect of corpus-based instruction on the grammatical accuracy of EFL learners in their use of past simple habitual expressions in writing were discussed. Participants’ grammatical accuracy performances were measured through written tests that included multiple-choice questions, gap-filling tasks, and paragraph writing. The frequency distribution and the error patterns associated with the above mentioned grammatical structures in the paragraph-writing tasks were examined.

The first research question was whether the experimental and the control groups significantly differed on their performance in multiple choice test as well as gap filling tests that were aimed at testing Past Simple habitual forms. The pre-test scores did not indicate statistically significant differences between the two groups which means that the initial proficiency was similar (Table 1). Nevertheless, the post-test results showed that it experienced a substantial positive gain compared to the experimental group (Table 2) which was explained by the corpus-based instruction that they were exposed to (Table 3). On the contrary, the control group which was taught through conventional means did not show any significant progress. These findings favor usage based theories like lexicogrammar and construction grammar that focus on learning on the basis of real and contextualized language intake [60,61]. The corpus-based teaching methodology concurs with these theories in that it exposes the learner to the real world patterns of language and as such, it promotes grammatical growth [62,63].

The second research question was based on the frequency distribution of Past Simple habitual forms (used to, would, and V2) in Ethiopian Students Corpus and the British National Corpus. The results showed that there is a considerable difference in the usage of these forms between Ethiopian learners and native speakers of English, with the latter using these forms more often, especially in affirmative situations (Fig 1). Ethiopian students were inclined to use more simple patterns and abuse some of the structures, particularly negative sentences. This tendency confirms the thesis made by [59] according to which EFL-students tend to overuse particularly taught grammatical structures because of a lack of natural input and the thesis of [64] that students prefer less complicated forms in non-immersive context. The reduced frequency of the complex habitual forms among Ethiopian learners is also very much indicating the exposure that is limited in the EFL settings (Larsen-Freeman and Long, 2014), whereas the prevalence of the interrogative forms could be associated with the practices in the classroom that are focused on drills [58]. In general, this suggests that the exposure and frequency of these effects are important to make the usage more native-like [59].

The third research question was concerned with the patterns of error in the use of past simple habitual expressions by the experimental and the control groups. Some mistakes that were common were the omission of subjects in clauses containing would or used to, the incorrect use of the -d ending of affirmative used to forms, use of use to in negative or interrogative clauses containing did, and the incorrect usage of would to singular past tense. Though both these mistakes were made in the two groups, errors in the experimental group were much fewer and this proves the usefulness of corpus-based instructions. This result promotes the function of input improvement in minimizing grammatical mistakes [65] and conforms to the studies that point to the complexity of tense-aspect structures [66,67]. In line with the previous studies, instruction based on the use of corpus enabled higher accuracy and better delineation of past habitual versus singular actions [55,68,69].

## Conclusions

This paper examined the effect of corpus based instruction on the grammatical accuracy of EFL learners with a special emphasis being placed on their use of past simple forms of habitual expression in writing. The pre-test findings showed that there was no significant difference between the experimental and the control group. Nevertheless, post-test data revealed that the experimental group has been significantly higher as compared to the control group. Such a result indicates that corpus-based training is more efficient as compared to the traditional one in terms of improving grammatical accuracy. It also enhances autonomy among learners and improves the confidence of learners.

On these findings, a number of conclusions can be made. The corpus-based instruction was a better method in enhancing the grammatical accuracy of EFL learners especially in application of the past simple habitual forms of speech in writing. Conventional grammar instruction which is mainly aimed at memorization and mechanical exercises did not seem to be very helpful. It did not adequately encourage learners or promote grammatical structure preservation in the long run. In comparison, corpus-based teaching is based on the real data of the language. It promotes learner independence and promotes better concept of grammar by the inductive and deductive processes.

This research also found that the experimental group taught with the help of a corpus-based teaching scored much higher after the testing than control group. This implies that frequency presentation and explicit grammar presentations in corpus materials make learners observe and internalize language patterns. As a result, students would be more precise in their usage of grammatical structure in writing. Comparatively, the control gave disorientation in the selection of correct grammatical forms and made more mistakes. Conventional strategies might therefore not be effective in tackling such grammar subjects.

## Supporting information

S1 File Frequency distribution of past simple tense (2). (XLSX)
